# Modular neuron-based body estimation: maintaining consistency over different limbs, modalities, and frames of reference

**DOI:** 10.3389/fncom.2013.00148

**Published:** 2013-10-28

**Authors:** Stephan Ehrenfeld, Oliver Herbort, Martin V. Butz

**Affiliations:** ^1^Cognitive Modeling, Department of Computer Science, Eberhard Karls University of TübingenTübingen, Germany; ^2^Department of Psychology, Julius-Maximilians UniversityWürzburg, Germany

**Keywords:** modular body schema, sensor fusion, multisensory perception, multisensory processing, multimodal interaction, probabilistic inference, population code, conflicting information

## Abstract

This paper addresses the question of how the brain maintains a probabilistic body state estimate over time from a modeling perspective. The neural Modular Modality Frame (nMMF) model simulates such a body state estimation process by continuously integrating redundant, multimodal body state information sources. The body state estimate itself is distributed over separate, but bidirectionally interacting modules. nMMF compares the incoming sensory and present body state information across the interacting modules and fuses the information sources accordingly. At the same time, nMMF enforces body state estimation consistency across the modules. nMMF is able to detect conflicting sensory information and to consequently decrease the influence of implausible sensor sources on the fly. In contrast to the previously published Modular Modality Frame (MMF) model, nMMF offers a biologically plausible neural implementation based on distributed, probabilistic population codes. Besides its neural plausibility, the neural encoding has the advantage of enabling (a) additional probabilistic information flow across the separate body state estimation modules and (b) the representation of arbitrary probability distributions of a body state. The results show that the neural estimates can detect and decrease the impact of false sensory information, can propagate conflicting information across modules, and can improve overall estimation accuracy due to additional module interactions. Even bodily illusions, such as the rubber hand illusion, can be simulated with nMMF. We conclude with an outlook on the potential of modeling human data and of invoking goal-directed behavioral control.

## 1. Introduction

Humans and other animals appear to learn and maintain a body schema^1^ (Graziano and Botvinick, 1999; Haggard and Wolpert, 2005), which is used to realize goal-directed behavior control. Evidence for having knowledge about the own body schema and associated body image is already found in 2-month old children, indicating that this knowledge is acquired very early in life (von Hofsten, 2004; Rochat, 2010). The more accurate the own body schema is, the more the infant is able to separate the external world (von Holst and Mittelstaedt, 1950) from its own body and, consequently, the more the infant is able to actively and goal-directedly explore the world (Konczak et al., 1995; Butz and Pezzulo, 2008). Developmental as well as neuroscientific evidence indicates that developing a body schema is critical for developing flexible, goal-directed behavioral control. In this paper we propose a computational neural model of how knowledge about the body can be represented, processed, and learned.

**Table 1:** **MMF-terminology**.

Body image	A usually conscious representation of the way the body appears from the outside (Haggard and Wolpert, 2005)
Body model	Static knowledge about the body: segmentation into body parts, metrics, and mappings between modules
Body schema	A group of body representations relevant for action (Haggard and Wolpert, 2005). This includes body state, body space, and body model. It allows for updates of the body state. The term body schema has been used across disciplines and with varying degrees of precision (Sekiyama et al., 2000; Buneo et al., 2002; Battaglia-Mayer et al., 2003; Maravita et al., 2003; Makin et al., 2008; Burns and Blohm, 2010; Hoffmann et al., 2010; Sober and Körding, 2012)
Body space	Teachable space of a particular body part in a particular modality
Body state	An estimate of the current body configuration. May refer to the body state encoded in a single module or spread over multiple modules
Distal-to-proximal	Mapping direction: fingertip → wrist → elbow → shoulder
Forward	Mapping direction: joint angles → local orientation → global orientation → location
Frame of reference	The coordinate system of a module: “global” (shoulder centered) or local (respective the next proximal body part)
Information fusion	Bayes optimal fusion of multiple probability distributions. These may include multiple sensors, multiple body states in different modules, or both
Inverse	Mapping direction: location → global orientation → local orientation → joint angles
Mappings	The set of connections between neurons in one or two input modules and neurons in one output module. There are three “types” of mappings: forward kinematics, inverse kinematics, and distal-to-proximal kinematics. They are used to propagate neuronal activity to other modules
Modality	Which information is encoded in which frame of reference: nMMF uses position-vectors, orientation-vectors in a “global” (i.e., respective the shoulder) or “local” (i.e., respective the next proximal body part) frame of reference, or joint-angles
Module	A state space of the body, such as the wrist location in space. Modules may differ with respect to modalities, frames of reference, and body parts
Neural population	A set of neurons that encode the spatial distribution in a particular module. The population as a whole encodes a probability distribution
nMMF	neural Modular Modality Frame model: the model presented in this work
Proximal-to-distal	Mapping direction: shoulder → elbow → wrist → fingertip (cf. Figure 4)
*q*^*i*^_*l*_	Probability mass of the l-th neuron in module i's population. The probability mass is the same as the Voronoi volume *V*_*l*_ (cf. Appendix A.2) times neuron l's probability density, normalized to 1
Sensor integration	The special case where sensory information is fused with the body state. Also, the result becomes the new body state
Transformation step	Projects input information from one or two modules to a neighboring module

When learning such a body schema, specific challenges must be met. First, sensory information about the body is available in different modalities and frames of reference. Thus, mappings between these modalities need to be established. Second, uncertainty due to noise, external forces, and changes of the body and the environment has to be handled effectively. Third, different information signals about the body may contradict each other, so that the maintenance of the present body state estimate is non-trivial.

The human brain has solved these challenges. In particular, the brain appears to be able to flexibly integrate multimodal sensory information about the body into a current estimate of its body state. This body state estimate seems to be modularized in two fashions: sensory modality-respective modularizations and body part-respective modularizations.

Evidence for sensor-specific modularizations can be found in brain imaging studies, which suggest that cross-modal sensory information fusion is common when perceiving the own body (Shams et al., 2000; Shimojo and Shams, 2001; Beauchamp, 2005). Related research suggests that body state representations are separated into body parts to certain degrees (Andersen et al., 1997; Gentner and Classen, 2006; Latash et al., 2007; Shadmehr and Krakauer, 2008; de Vignemont et al., 2009). Thus, a highly modularized body state estimate is maintained by our brain.

For maintaining such a modularized but consistent body state estimate, information is effectively interchanged and fused across the modularizations (Tononi et al., 1998; Ernst and Bülthoff, 2004; Stein and Stanford, 2008). Hereby, the information exchange typically depends on how the body is currently positioned and oriented in space (Holmes and Spence, 2004; Butz et al., 2010). Neurological disorders further indicate that both sensory input and body state estimates are fused across modules (Giummarra et al., 2008). To combine incoming sensory information with the most accurate body state estimate, the brain also anticipates body state changes and consequent sensory feedback during movement execution (von Holst and Mittelstaedt, 1950; Blakemore et al., 2000; Sommer and Wurtz, 2006). Many of these interactions seem to take place in early stages of the cortical processing hierarchy (Stein and Stanford, 2008), probably before the sensory information is fully integrated into the own body state estimate. Further evidence for sensory information comparisons and the flexible fusion of this information for maintaining body state estimates is given by multimodal illusions like the rubber hand illusion (Botvinick and Cohen, 1998; Haggard and Wolpert, 2005; Makin et al., 2008) and the Pinocchio illusion (Lackner, 1988). Thus, it appears that while the brain's body state estimate is highly modularized, many interactions ensure an effective estimate maintenance and sensory information integration. However, it remains unclear how, when, and which information is compared and selectively fused.

We recently proposed the Modular Modality Frame (MMF) model (Ehrenfeld and Butz, 2011, 2012, 2013), which models the maintenance of a body state estimate given noisy, multimodal sensory information sources. The MMF model fully relies on hard-coded kinematic knowledge of the simulated body and estimates body states by means of Gaussian probability densities. Here we present a neural extension of MMF—the neural Modular Modality Frame (nMMF) model. The novel contributions of nMMF are as follows:

First, body spaces, current body state estimation modules, and mappings between body modules are now implemented neurally. As a result, nMMF is able to encode arbitrary, even multimodal body state estimations. Moreover, the neural population encodings for body state estimates are plausible from a computational neuroscience perspective (Deneve and Pouget, 2004; Knill and Pouget, 2004; Denève et al., 2007; Doya et al., 2007). Second, we now ensure that the Shannon entropy of a distribution remains unchanged during multi-body state fusion, in order to avoid excessive information gain when fusing dependent sources of information. Third, information exchange is no longer restricted to forward and inverse kinematic mappings. Distal-to-proximal mappings are also included. This means that information about the hand in space can, for example, influence the estimate of the elbow location, of the orientation of the upper arm, or even of the shoulder joint angles.

The remainder of this paper is structured as follows. First, the nMMF model is detailed. Next, nMMF is evaluated on a simulated two degree of freedom arm in a two-dimensional setup. The evaluations show that nMMF is able to detect faulty sensory information on the fly and is able to propagate information appropriately distal-to-proximal, i.e., from hand to upper arm. In the final discussion, we compare nMMF to related models and sketch-out future research directions.

## 2. Materials and methods

nMMF is inspired by those processes of human body state estimation which are detailed above. In a computational framework, these processes can be approximated by five key assumptions: (1) the body state is continuously estimated probabilistically over time; (2) multimodal, redundant sensory information sources are integrated based on Bayesian principles; (3) the body state representation is modularized along body parts as well as along modalities and their corresponding frames-of-reference; (4) the body modules are locally interactive in that information about the body state is compared and fused locally; (5) the redundant, modularized representation of the body is exploited for autonomous sensor failure detection and subsequent avoidance of the failing sensor's influence.

We now detail how these key aspects are realized in nMMF. First, we describe which modules are used, second, how neurons encode the sensory inputs and the body state, third, how information is fused, fourth, how information is projected across modules, fifth, how conflicting information is detected and blocked out, and, finally, how the overall information flow unfolds over time. In the subsequent evaluation section we show how nMMF processes sensory information, how faulty sensory information can be ignored to a certain degree, but also how such faulty sensory information can influence the complete body state estimation.

### 2.1. Modules

nMMF represents a body state by a collection of modules, where each module represents an aspect of the overall body state. In particular, nMMF's modules differ with respect to (1) the encoded joint (or the next distal limb) and (2) the modality frame in which the joint or limb is encoded. The term modality frame defines which modality is perceived (location, orientation, or joint angle) and in which frame of reference the modality is encoded (shoulder-centered or “local” with respect to the next proximal limb).

In the following, we focus on a general description of a humanoid arm, although the same principle may apply for a complete body description. First, we specify the state of an arm in general. Next, we detail how nMMF encodes the arm state in its respective modules.

#### 2.1.1. Arm specification

**Figure 1:**
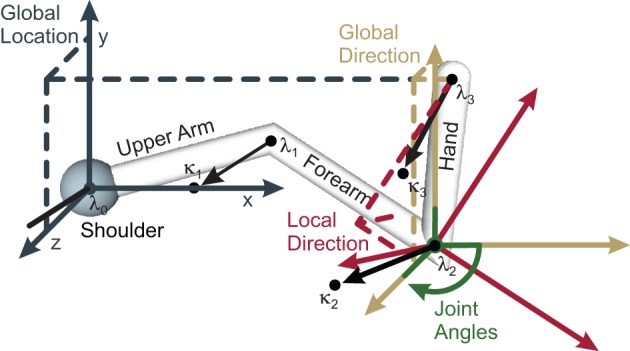
**Schematic of the four “hand”-limb-encoding modules**. Three coordinate systems (solid axes) are shown, together with the components (dashed lines) of the respective encoded vector. Dark gray (Global Location module): the coordinate system is centered around the shoulder with fixed orientation. Encoded is the global location vector, which goes from shoulder to the end-effector. Yellow (Global Orientation module): the coordinate system has the same orientation as the gray one but in this case the limb orientation is encoded by the means of two vectors: a unit vector parallel to the “hand” limb (shown, dashed lines), and a perpendicular vector (not shown). Red (Local Orientation module): the local coordinate system is oriented along the forearm. Relative to this forearm orientation, the orientation of the “hand” limb is encoded—by a unit vector parallel to the “hand” limb (shown), and a perpendicular vector (not shown). Green (Local Angle module): the fourth module encodes angles. The same four modules and respective coordinate systems exist for the forearm and the upper arm (not shown). Modified based on Ehrenfeld and Butz (2012, 2013).

An arm state may be encoded by the arm's location in space, its limb orientations, or the joint angles. With respect to the arm's location, we denote the shoulder (elbow, wrist, fingertips) location by **λ**_0_ (**λ**_1_, **λ**_2_, **λ**_3_) (cf. Figure 1 for an illustration). To derive the arm limb orientations we simply subtract successive limb locations. To additionally encode the inner rotations of the respective limbs, we define a point **κ**_*i*_ for each limb *i*, where **κ**_*i*_ is locked relative to the limb. Essentially, **κ**_*i*_ always lies somewhere on the unit circle around **λ**_*i*_, where the unit circle's plane is perpendicular to the orientation of limb *i*. Finally, the joint angles of each arm joint *i* are denoted by the Tait-Bryan angles (ϕ_*i*, 1_, ϕ_*i*, 2_, ϕ_*i*, 3_), which rotate about the intrinsic rotation axes ^*i*−1^***x***, ^*i*−1^***y***′, ^*i*−1^***z***″, where one (two) apostrophes denote that the rotation axis has been rotated by the angles ϕ_*i*, 1_ (and ϕ_*i*, 2_).

#### 2.1.2. nMMF's arm encoding

nMMF encodes probabilistic arm states by means of distributed population codes in redundant modules. In particular, each limb is encoded in four modality frames: global location (*GL*), global orientation (*GO*), local orientation (*LO*), and local (joint) angles (*LA*). Note that other modalities could be used in addition and other combinations of modalities and frames of reference are possible—such as a local location. It is crucial, however, that the chosen combinations form a redundant estimate of the overall body state. nMMF's implemented modules and their interactions are shown in Figure 4; Figure 1 shows the employed modality frames for an exemplar arm.

To encode each modality frame, respective coordinate systems need to be defined. In order to provide a consistent notation for all nMMF modules, we introduce **x**^*Z*_*i*_^ as the estimated arm state of limb *i* in modality frame *Z*, where *Z* ∈ {*GL, GO, LO, LA*}^2^.

The first modality frame encodes the global location (*GL*) of an arm limb. Limb *i*'s end point **λ**_*i*_ in the *GL* modality frame is the 3D vector from the shoulder to the end-point of limb *i*:

(1) $$x^{GL_{i}}\equiv \lambda_{i}-\lambda_{0}.$$

The global orientation (*GO*) is a 6D vector. It concatenates both a 3D unit-vector in the direction of the arm limb, and a 3D unit-vector perpendicular to the arm limb dependent on its inner rotation:

(2) $$x^{GO_{i}}\equiv \left(\begin{array}{c} \text{unit}\left(\lambda_{i}-\lambda_{i-1}\right)\\ \text{unit}\left(\kappa_{i}-\lambda_{i-1}\right) \end{array}\right),$$

As both vectors are unit vectors and are perpendicular to each other, three degrees of freedom are canceled out and all remaining orientation vectors form a 3D manifold in 6D space.

The local orientation (*LO*) is analogous, but expresses both subvectors in a local coordinate system (e.g., *LO*_2_ is expressed in a coordinate system whose axes are defined by *GO*_1_). Again, only a 3D manifold remains:

(3) $$x^{LO_{i}}\equiv \left(\begin{array}{c} \text{unit}\left({}^{i-1}\lambda_{i}{-}^{i-1}\lambda_{i-1}\right)\\ \text{unit}\left({}^{i-1}\kappa_{i}{-}^{i-1}\lambda_{i-1}\right) \end{array}\right).$$

Note that we use the pre-superscript to denote a particular, relative coordinate system, whereas we use the subscript to denote a particular limb. Furthermore, note that ^*i*−1^**λ**_*i*−1_ ≡ (0, 0, 0)^*T*^ due to the definition of the coordinate system relative to limb *i* − 1.

Finally, the local angles (*LA*) are encoded as Tait-Bryan angles

(4) $$x^{LA_{i}}\equiv {\left(\phi_{i,1},\phi_{i,2},\phi_{i,3}\right)}^{T},$$

which is identical to the arm encoding itself.

Note that all modality frames are maximally 3D. Thus, the locality of the modular architecture ensures that the amount of neurons needed to represent a particular modality frame with a neural population code of *n* neurons per dimension scales in *O*(*n*^3^).

### 2.2. Probabilistic representation

In complex tasks, uncertainty is ubiquitous due to sensory and motor noise, external forces, changes in the environment, and changes of the body schema. To deal with this uncertainty, humans apply probabilistic body state estimations (Ernst and Banks, 2002; Körding and Wolpert, 2004). In computational models (e.g., Ma et al., 2006), state estimates are often simplified by confining probability density estimates to one type of distribution (such as the Gaussian, Gamma or Poisson distributions). However, shapes may vary greatly due to non-linear influences of mappings across modules, constraints (like joint restrictions or obstacles), varying shapes of sensory input to begin with, or even neural disorders. Moreover, in certain circumstances the brain may actually maintain multimodal alternatives about the current body state.

In contrast to MMF, nMMF approximates probability distributions with neural population codes (Deneve et al., 1999) to enable the representation of probability distributions with arbitrary shapes. Each neuron in such a code is responsive to specific values of the input data (preferred value) and thus has a local receptive field of a particular size. Note that by using population codes, the shapes of the encoded probability distributions become unconstrained. The modularity of nMMF ensures a scalable neural encoding of the arm or even the full body. In the following, we describe how the receptive fields and the preferred values of the population neurons are determined.

#### 2.2.1. Sampling of neural populations

In order to create neurons only within the reachable manifolds, we let the populations of neurons grow while observing simulated arm states. This is done in the following way: A simulated arm is set to a random arm position, which is uniformly distributed in angular space. Then, noiseless measurements **z**^*j*^ are obtained in each module *j*. If

(5) $$||z^{j}-x_{l}^{j}||>d_{\text{min}}\forall l\in \{1,\ldots ,N^{j}\},$$

a new neuron is added at **z**^*j*^, where **x**^*j*^_*l*_ denotes the preferred value of neuron *l* and *N*^*j*^ the current number of neurons that exist in module *j*. Next, the arm is set to a new random position. Thus, all sampling positions are independent of each other and the resulting neurons in each module are approximately uniformly distributed, covering the reachable manifold.

#### 2.2.2. Tuning function

Each neuron has an associated tuning function (Deneve et al., 1999), which specifies how the neuron responds to a signal. We use Gaussian tuning functions with mean **x**_*l*_ and covariance **R**. For instance, if a measurement signal occurs at position **z**, the probability density function (PDF) at **x**_*l*_ is:

(6) $${\text{p}}_{l}=N\left(z,R\right)\left(x_{l}\right).$$

**Figure 2:**
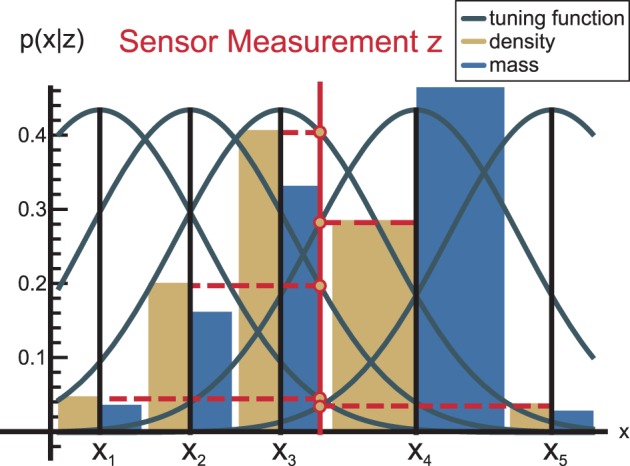
**Each neuron has a tuning function (Deneve et al., 1999) that defines how the neuron responds to a signal**. Generally, these tuning functions are considered to be bell-shaped, such as the shown Gaussian kernels. As a consequence of this encoding, the PDF encoded by the neural population becomes Gaussian as well (yellow bars), while the probability mass (blue) is somewhat distorted because it accounts for the local neural density.

In effect, a Gaussian PDF is activated over the whole neural population (cf. Figure 2, yellow bars for an illustration). If the covariance **R** of all tuning functions is equal to the sensor covariance, then Equation (6) is the same as the inverse measurement model (Thrun et al., 2005).

Since probability mass has to be conserved when information flows from one module to another in nMMF, we derive the probability mass function (PMF) from the PDF. Note that the neural PMF encoding will typically slightly differ from the PDF encoding in nMMF, because the population codes in nMMF may not be uniformly distributed. This is illustrated in Figure 2.

#### 2.2.3. Probability mass

Let **X** be a multivariate random variable, and ω a subset of a sample space Ω. The probability mass *q* in ω corresponds to the probability that *X* lies in ω:

(7) $$q_{\omega }\equiv Pr\left[X\in \omega \right]=\int_{\omega }\text{p}\left(x\right)dx$$

Just as *N* neurons are spread over Ω, Ω is discretized into *N* subsets ω_*l*_, *l* ∈ (1..*N*), which are simply the Voronoi cells *R*_*l*_ of those neurons (cf. Appendix A.2). The probability mass of a neuron can then be approximated by the Volume *V* of the cell times the density (Equation 6) at the neuron's position

(8) $$q_{l}=\int_{R_{l}}\text{p}\left(x\right)dx\approx \frac{V_{l}\cdot \text{p}\left(x_{l}\right)}{\sum_{l^{*}=1}^{N}V_{l^{*}}\cdot \text{p}\left(x_{l^{*}}\right)},$$

where the denominator normalizes the probability mass to 1. An illustration of a probability mass is shown in Figure 2, blue bars. To handle potential approximation errors, we ensure that the sum of the probability mass over all neurons *N* in a module is always normalized to 1, by

(9) $$q_{l}\leftarrow \frac{q_{l}}{\sum_{l^{*}}q_{l^{*}}},$$

where the symbol “←” is used as a value update assignment.

### 2.3. Information fusion

With a neural, modularized, probabilistic body state representation in hand, we now focus on information processing and information exchange. In this section, we first detail the fusion of different neurally-represented PDFs, and consecutively derive the fusion of different PMFs. Two cases are considered: that the information carried by the different PMFs is dependent or independent.

The Bayesian fusion (Bloch, 1996) of multiple independent neurally-encoded probability distributions is the neuron-wise product of the respective PDFs. Thus, the fusion yields:

(10) $${\text{p}}_{\text{fused},l}\propto \prod_{j=1}^{M}{\text{p}}_{j,l},$$

where *M* specifies the number of modality frames that are fused, *l* is the index of a specific neuron, and p_*j, l*_ encodes the probability density that stems from modality frame *j* and that is covered by neuron *l*. As the density can be converted to a mass by p_*l*_ = *q*_*l*_ · *V*^−1^_*l*_, applying this identity to both sides of Equation (10) yields the fusion of PMFs

(11) $$q_{\text{fused},l}=\frac{{\left(V_{l}\right)}^{-(M-1)}\prod_{j=1}^{M}q_{j,l}}{\sum_{l^{*}}{\left(V_{l^{*}}\right)}^{-(M-1)}\prod_{j=1}^{M}q_{j,l^{*}}}.$$

When Equations (10) or (11) is used to fuse partly or fully dependent information, the resulting distribution is overconfident (i.e., too narrow).

**Figure 3:**
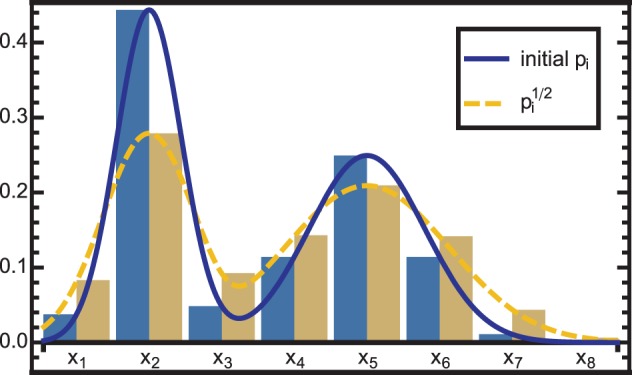
**The solid blue curve is modified by raising the PDF to the power of $\frac{1}{2}$ neuron-wise, resulting in the dashed yellow curve**. As the exponent is <1, the distribution is widened, i.e., information is diffused. This effect is used in two cases: (1) to correct for overconfidence due to the combination of dependent information sources and (2) to reduce the influence of a module that is in conflict with other modules.

To correct for this overconfidence, the PDF can be raised to the power of an exponent α < 1. However, since we encode PMFs, additional conversions are again necessary to account for the Voronoi volumes covered by the respective neurons. The correction for overconfidence is thus accomplished by:

(12) $$q_{\text{fused},l}\leftarrow \frac{V_{l}^{1-\alpha }{\left(q_{\text{fused},l}\right)}^{\alpha }}{\sum_{l^{*}}V_{l^{*}}^{1-\alpha }{\left(q_{\text{fused},l^{*}}\right)}^{\alpha }};$$

where the denominator normalizes the mass to 1. The effect is a widening of the encoded PMF, which is illustrated in Figure 3.

To infer the exponent α, a measure of information content is required. We use the Shannon entropy *h* to estimate the amount of information in a PMF:

(13) $$h\equiv -\sum_{l}q_{l}\cdot \ln\left(q_{l}\right),$$

where *q*_*l*_ may denote the fused distribution as in Equation (11) or any other arbitrary distribution. If all distributions were Gaussian, the exponent could be derived from Equation (12) by requiring that the Shannon entropy in a module before fusion should be equal to the Shannon entropy after fusion:

(14) $$\alpha ={\text{e}}^{-2\left(\min_{j}h_{j}-h_{\text{fused}}\right)}.$$

Due to the lack of a rigorous derivation of α in the general case, we utilize this approximation to determine α for our population-encoded probability masses in each module.

### 2.4. Cross-module connections

With notations for modules in nMMF, neurally-encoded probability masses, and information fusion of redundant sources of information at hand, we now specify how the neural, cross-module connections are implemented in nMMF.

**Figure 4:**
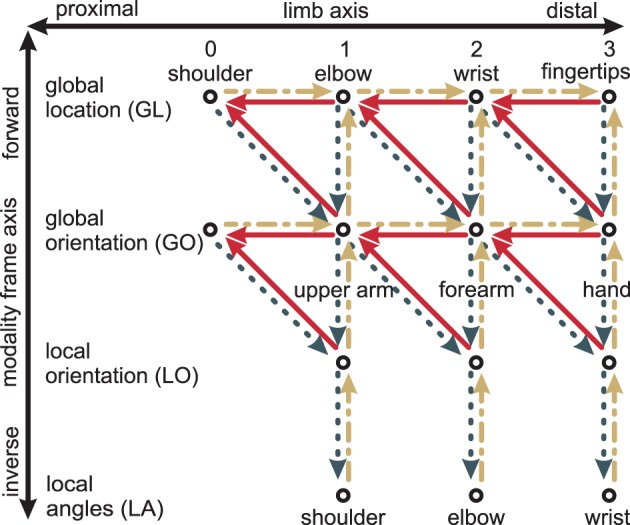
**Transformation steps between different modules: The modules (shown as circles) differ with respect to limbs (horizontal axis) and with respect to modalities and frames of reference (vertical axis)**. Every transformation step consists of one or two input modules and one output module. An example is the two solid lines on the top right: together, they encode how the wrist location *GL*_2_ depends on both the fingertip location *GL*_3_ and the global hand orientation *GO*_3_. Yellow dash-dotted lines are the forward kinematics, dark gray dotted lines the inverse kinematics, and red solid lines the distal-to-proximal kinematics. Modified based on Ehrenfeld and Butz (2012, 2013).

Modules may differ along two axes: the limb-axis (proximal-to-distal, shown horizontally in Figure 4), and the modality frame axis (forward and inverse, shown vertically in Figure 4). Information may flow from one or two input modules to a neighboring output module. This may happen diagonally: Out of the four diagonal directions, only three are single transformation steps: proximal-to-distal-forward, proximal-to-distal-inverse, and distal-to-proximal-forward.^3^ Together, all three form a triangle in Figure 4—e.g., (*GL*_2_, *GL*_3_, *GO*_3_). In robotics, proximal-to-distal-forward and proximal-to-distal-inverse are typically termed forward and inverse kinematics, respectively, while distal-to-proximal mappings are often ignored.

#### 2.4.1. Single transformation steps

Rather than learning the neural connections, here we use hard-coded kinematic mappings

(15) $$x^{j,k\to i}\left(m,n\right)=f^{j,k\to i}\left(x_{m}^{j},x_{n}^{k}\right),$$

where *i, j, k* are neighboring modules of nMMF. A derivation of the closed form of **f**^*j, k* → *i*^ can be found in Ehrenfeld and Butz (2013).

For all pairs of input neurons *m* and *n*, connections are built to those neurons *l* in the output module, which are sufficiently close to the transformation result **x**^*j, k* → *i*^(*m, n*). The Gaussian (Equation 30) value for the Euclidean distance of each neuron *l* in the output module *i* to the transformation result **x**^*j, k* → *i*^(*m, n*) is used as connection strength *w*:

(16) $$w_{m,n\to l}^{j,k\to i}=V_{l}\cdot N\text{​}\left(x^{j,k\to i}\left(m,n\right),R_{\text{Map}}^{i}\right)(x_{l}^{i}),$$

where the receptive field covariance **R**^*i*^_Map_ regulates how much the mapping itself widens the encoded probability distribution. It models an information loss during a transformation, either due to inaccurate mappings or due to discretization errors. Since we use accurate mappings, we only need to consider the latter and therefore base **R**^*i*^_Map_ on the neuron distance in the output module.

If the transformation step has two inputs from the location modality *GL* (e.g., an elbow location *GL*_1_ and a wrist location *GL*_2_) the distance of both neurons' preferred values |**x**^*GL*_2_^_*m*_ − **x**^*GL*_1_^_*n*_| must be approximately equal to the length of the forearm. We introduce a modifying factor *F* with respect to neurons *m* and *n*, which reflects how well the constraint is met:

(17) $$F_{mn}={\text{e}}^{-\frac{1}{2}\frac{\Delta x_{mn}^{T}}{\left|\Delta x_{mn}\right|}\text{​}{\left(R_{\text{Map}}^{i}\right)}^{-1}\text{​​}\frac{\Delta x_{mn}}{\left|\Delta x_{mn}\right|}\cdot {\left(\left|\Delta x_{mn}\right|\text{​}-\text{​}d_{\text{limb}}\right)}^{2}}\text{​​},$$

where *d*_limb_ is the length of the respective arm limb, and Δ**x**_*mn*_ ≡ **x**^*GL*_2_^_*m*_ − **x**^*GL*_1_^_*n*_ the relative position of both input neurons. Intuitively, (|Δ**x**_*mn*_| − *d*_limb_)^2^ results in a penalization of larger deviations from the limb length, and the first factors scale this penalization dependent on the covariance in the mapping. For all other transformation steps, no constraints are necessary, and *F*_*mn*_ = 1 in these cases. In consequence, the connection weights *w* are normalized by

(18) $$w_{m,n\to l}^{j,k\to i}\leftarrow \frac{F_{mn}\cdot w_{m,n\to l}^{j,k\to i}}{\sum_{l^{*}}w_{m,n\to l^{*}}^{j,k\to i}}\;\forall m,n,$$

where the modifying factor *F*_*mn*_ blocks the influence of pairs of location neurons that do not correspond with the arm length sufficiently well.

Finally, the projection of two probability distributions *q*^*j*^, *q*^*k*^ along the connections *f*^*j, k* → *i*^ into module *i* yields

(19) $$q_{l}^{i}=\frac{\sum_{m}\sum_{n}q_{m}^{j}q_{n}^{k}w_{m,n\to l}^{j,k\to i}}{\sum_{l^{*}}\sum_{m}\sum_{n}q_{m}^{j}q_{n}^{k}w_{m,n\to l^{*}}^{j,k\to i}},$$

where the denominator normalizes the overall activity again to 1.

#### 2.4.2. Chain of transformation steps

As nMMF's modules are strongly interconnected, information flows from any module to all other modules. This requires that multiple information transformation steps be done successively.

In nMMF, information is projected into other modules by means of two different approaches. The first approach is used when information needs to stay independent for determining plausibility estimates (cf. section 2.5). In this case, the forward or inverse kinematic mappings are used without fusing other information on the way. Thus, information is not mixed and projections of independent information sources into a common module stay independent. For example, sensory input from a local angle module may be projected to the corresponding global location module by the forward kinematics chain *LA* → *LO* → *GO* → *GL*. Meanwhile, sensory information from the global orientation may also be projected into *GL* by *GO* → *GL*. These two information sources remain independent of each other but are now represented in a common module and can thus be directly compared.

The second approach is used when information is fused across modules (cf. section 2.6). In this case, the information is projected across the modules of nMMF by alternating between local projection and information fusion steps. For example, the *LA* information is projected to *LO*, where the result is fused with the *LO* input. The fused result is then projected further to *GO*, where the result is fused again, and so on. This method enables the integration of even incomplete information^4^ and it reduces computation time because fewer transformation steps are required.

### 2.5. Conflict resolution

The information, which is exchanged via the specified cross-module connections, has a specific certainty to it. This certainty is encoded implicitly in the neural population codes in each module. Sensory signals are encoded in a population code by making assumptions about the noise in the signal, typically using a measurement model (Thrun et al., 2005). However, those assumptions can be violated by, for example, sudden occurrences of systematic sensor errors, unacquainted environmental conditions, or changes in the body schema due to growth or injury. To be able to account for such potentially unknown signal disturbances, nMMF estimates plausibilities for each signal. If a signal has low plausibility, it is mistrusted and its information content is consequently decreased.

Because the true state of the body is unknown, nMMF estimates signal plausibilities by comparing different, redundant information sources. The modular encoding of the body in nMMF is highly suitable for conducting such comparisons. Given several redundant distributions about a body state, a failing distribution can be detected when it systematically and strongly differs from the complementary, redundant sources of information.

#### 2.5.1. Acquisition of plausibilities

Let *m*_12_ be a measure of how well two sources (or distributions) 1 and 2 match each other. Zhang and Eggert (2009) provide an overview of different potential measures for *m*_12_. In nMMF, we use the scalar product as a matching measure. Given any neural module *i*, in which two PMFs (1 and 2) are encoded, their relative match is determined by:

(20) $$\begin{array}{l} m_{12}^{i}=\frac{q_{1}^{i}\left(x^{i}\right)\cdot q_{2}^{i}\left(x^{i}\right)}{\left|\left|q_{1}^{i}\left(x^{i}\right)\right|\right|\cdot \left|\left|q_{2}^{i}\left(x^{i}\right)\right|\right|}\\ =\frac{\sum_{l}q_{1,l}^{i}\cdot q_{2,l}^{i}}{\sqrt{\sum_{l}q_{1,l}^{i}\cdot q_{1,l}^{i}}\cdot \sqrt{\sum_{l}q_{2,l}^{i}\cdot q_{2,l}^{i}}}, \end{array}$$

where the dot · in the first line's numerator is the inner product of the two functions *q*^*i*^_1_(**x**^*i*^) and *q*^*i*^_2_(**x**^*i*^). The measure *m*^*i*^_12_ is symmetric, i.e., *m*^*i*^_12_ ≡ *m*^*i*^_21_. Thus, if one source has an offset, the matching measure can not determine which of the two sources has that offset. This can be solved by comparing multiple pair matches given at least three redundant sources of information.

To identify faulty sensory information, nMMF computes a plausibility value *m*^*i*^ for each information source *i* by comparing it to multiple other redundant information sources *j*. The most direct comparison is done by determining the mean of the matches of channel *i* with all other channels *j*, whose information was transferred to module *i*:

(21) $${\left(m^{i}\right)}^{*}=\frac{1}{N-1}\sum_{j=1,j\neq i}^{N}m_{ij}^{i}.$$

The measure may be termed an absolute plausibility measure of information source *i*. To obtain the final plausibility value, the relative matching quality is determined by dividing (*m*^*i*^)^*^ by the highest absolute plausibility measure (*m*^*j*^)^*^ of all related sources:

(22) $$m^{i}=\frac{{\left(m^{i}\right)}^{*}}{\max_{j}{\left(m^{j}\right)}^{*}}.$$

**Figure 5:**
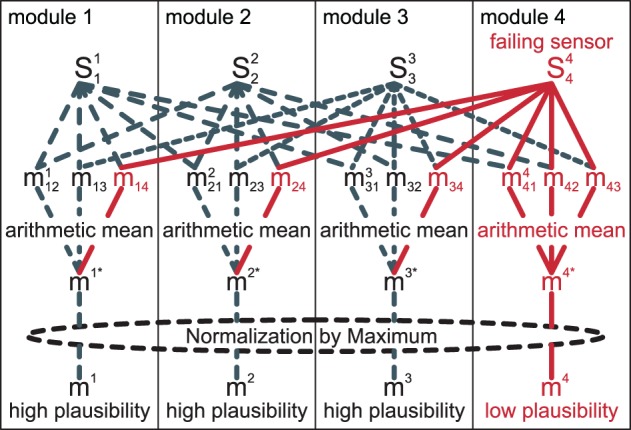
Matches *m*^*i*^_*ij*_ for pairs of two sources are obtained, then an arithmetic mean over all *j* yields (*m*^*i*^)^*^. Finally, a normalization by the maximum of all (*m*^*j*^)^*^ yields the final plausibility *m*^*i*^.

The whole process is illustrated in Figure 5. In the illustration, sensor *S*^4^_4_ is assumed to have a systematic error. As the sensor is always included for comparisons in its own module *m*^4^, but only once in each other module, the arithmetic mean of its matching value is lower than that of the others. In our experience, this approach of comparing pairs of information sources is more robust than, for example, comparing one sensor to the combined information of all other sensors.

In summary, if a channel *i* is in accordance with most of the other channels, the plausibility estimate *m*^*i*^ will be relatively high. In contrast, if a specific channel *i* systematically deviates from all other channels, its plausibility estimate will be relatively low.

#### 2.5.2. Usage of plausibilities

To incorporate the plausibility estimates into the sensor fusion process, the contribution of each information source *i* is weighted by its plausibility estimate *m*^*i*^. This is done by Equation (12), where the exponent α^*i*^ needs to depend on the plausibility *m*^*i*^. Boundary constraints are α^*i*^(0) = 0, α^*i*^(1) = 1 and the mapping should strictly increase monotonically. We simply set α^*i*^ ≡ *m*^*i*^, which meets these constraints.

### 2.6. Interactive information flow

**Figure 6:**
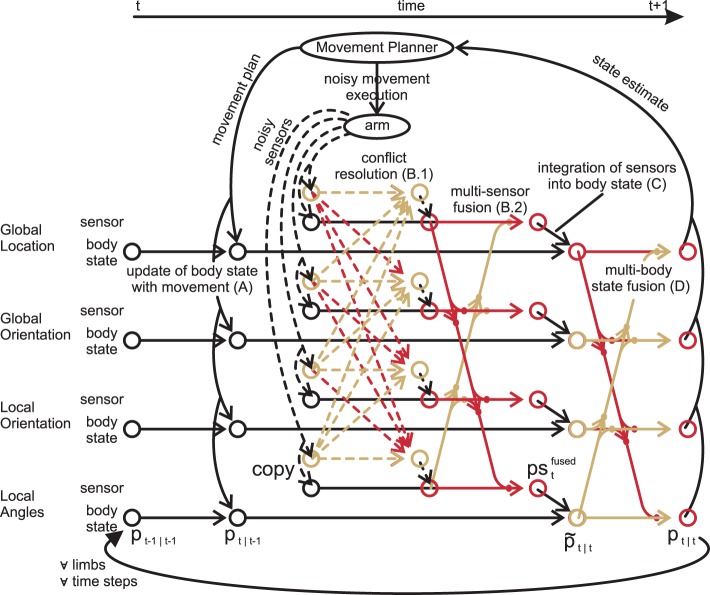
**Data flow for one limb: for simplicity, the inter-limb dependencies are not shown**. First, the forward model predicts the state estimate after the movement **(A)**. Second, the measurements are transformed from all modality frames to all other frames (dashed lines), where their respective qualities are calculated **(B.1)**. Third, copies of the original measurements are fused weighted with both the quality and the quantity of their information **(B.2)**. These fused measurements are then integrated in their respective modality frame **(C)**. Lastly, the crosstalk shifts all state estimates toward all other estimates, synchronizing them **(D)**. **(A–D)** are then repeated for other limbs and other time steps. Modified based on Ehrenfeld and Butz (2012, 2013).

With all options for information fusion at hand, we can finally specify the iterative information flow in nMMF. nMMF maintains an arm state estimate over time by executing four processing steps in each time step: a prediction step (A), a sensor fusion step (B), an update step (C), and a crosstalk step (D) (cf. Figure 6). The prediction step includes the impact of the movement on the estimates. The sensor fusion step first increases the dispersion of those sensory distributions that badly match other sensors. After that, the modified sensory distributions are fused. The next step integrates the sensor fusion result into the estimate of the body state. The last step enforces synchronization between the individual modules of the body state.

#### 2.6.1. Prediction step

In order to be able to use the information from previous time steps, the impact of any movement of the arm on the state estimates *q*^*i*^(**x**) is predicted. First, the arm movement Δ**y** and motor noise **P**_Δ**y**_ are projected from motor space to all nMMF modules by linear approximations, resulting in Δ**y**^*i*^ and **P**^*i*^_Δ**y**_. The involved Jacobians can be found in Ehrenfeld and Butz (2013).

Second, the impact of the movement is predicted by convolving the probability distribution of the last time step *q*^*i*^_*t*−1|*t*−1_(**x**^*i*^) with the Gaussian *N*(Δ**y**^*i*^,**P**^*i*^_Δ**y**_). This convolution can be understood as a translation of *q*^*i*^_*t*−1|*t*−1_(**x**^*i*^) along the vector Δ**y**^*i*^ and a blurring with the covariance **P**^*i*^_Δ**y**_. Thus the activity *q*^*i*^_*n*_ of some source neuron *n* in module *i* flows to all target neurons *l* in the same module. The consequent *a priori* activity of target neuron *l* after movement but before any sensor consideration can be determined by:

(23) $$q_{l,t|t-1}^{i}\leftarrow \;\sum_{n}(q_{n,t-1|t-1}^{i}\cdot \frac{V_{l}N\text{​}\left(x_{n}^{i}+\Delta y^{i},P_{\Delta y}^{i}\right)\left(x_{l}\right)}{\sum_{l^{*}}V_{l^{*}}N\text{​}\left(x_{n}^{i}+\Delta y^{i},P_{\Delta y}^{i}\right)\left(x_{l^{*}}\right)}),$$

where the derivation is specified in the Appendix, cf. Equation (31). The equation sums up the activities from all source neurons *n*, where *N* is the Gaussian, which does the translation and blurring. The normalization in the denominator ensures that the activity that flows from each source neuron *n* is preserved.

#### 2.6.2. Multi-sensor fusion

During multi-sensor fusion, conflicting information content is reduced by deriving sensory plausibilities for each module (Equation 22) and modifying the sensory inputs using (Equation 12). Second, the modified distributions are projected across modules (Equation 19) in order to provide each module with all the sensory input. During this projection, chains of transformation steps accumulate information from more and more modules along the way. Finally, in each module *i*, the underlying distribution is fused with the outputs from all three chains (forward, inverse, and distal-to-proximal). With Equation (11) the fusion is:

(24) $$s_{l,t}^{i,\text{fused}}=\frac{V_{l}^{-3}s_{l,t}^{i}\cdot s_{l,t}^{i}|\text{for}\cdot s_{l,t}^{i}|\text{inv}\cdot s_{l,t}^{i}|\text{dis}}{\sum_{l^{*}}V_{l^{*}}^{-3}s_{l^{*},t}^{i}\text{​}\cdot \text{​}s_{l^{*},t}^{i}|\text{for​}\cdot \text{​}s_{l^{*},t}^{i}|\text{inv​}\cdot \text{​}s_{l^{*},t}^{i}|\text{dis}},$$

where the notation |xyz is used to indicate the particular sensory information source that is projected into module *i* and *s*^*i*^_*l, t*_ denotes neuron *l*'s share of this information^5^. The denominator normalizes the result.

#### 2.6.3. Sensor integration

After sensor fusion, the fused sensor distributions *s*^*i*, fused^_*l, t*_ (Equation 24) are fused again, but this time with the *a priori* state estimate distributions *q*^*i*^_*l, t*|*t*−1_ resulting from the prediction step (Equation 23). The resulting posterior distribution before the final crosstalk step (denoted by ~) thus equates to:

(25) $${\tilde{q}}_{l,t|t}^{i}=\frac{V_{l}^{-1}q_{l,t|t-1}^{i}\cdot s_{l,t}^{i,\text{fused}}}{\sum_{l^{*}}V_{l^{*}}^{-1}q_{l^{*},t|t-1}^{i}\cdot s_{l^{*},t}^{i,\text{fused}}}.$$

#### 2.6.4. Multi-body state fusion

Finally, the module interaction in nMMF is applied to ensure that the state estimates stay consistent across the modules. This is done the same way as in multi-sensor fusion, except that afterwards the resulting distributions are modified such that each one has the same entropy as it had before (using Equations 12–14). Thus, during multi-body state fusion, information is first erroneously gained, and then corrected for by artificial information loss. The crosstalk step essentially shifts the means and shapes of each distribution toward other modules, ensuring consistency over modules. It does so without changing the distribution width. As a result, we have determined the final posterior distribution encoded by the probability masses in all neurons *l* for all modules *i*, denoted by *q*^*i*^_*l, t*|*t*_.

This step concludes the iterative information processing in nMMF, which continuously cycles over these processing (cf. Figure 6) steps over time. In the following, we validate the functionalities and capabilities of nMMF.

## 3. Results

To test if nMMF is capable of maintaining a coherent body state estimate, we evaluated nMMF in a simple arm model setup, in which a simulated sensor failure occurs temporarily. We then analyzed whether the sensor failure can be detected (section 3.2); whether the sensor failure can be compensated for (section 3.3); how the available, partially conflicting information is propagated across modality frames (section 3.4); and if the distal-to-proximal mappings improve nMMF's state estimation (section 3.5).

### 3.1. Arm setup

To keep it simple, we use a minimally complex arm, which still shows all essential characteristics (i.e., modules that differ with respect to modalities, frames of reference and limbs, and cross-module interactions as in section 2.6). Specifically, a simulated planar arm with two limbs is used. The arm is controlled by a kinematic simulator, disregarding angular momentum or gravity. The simulator executes noisy movements with mean zero in the (x,y)-plane. The motor noise in the angular modules is

(26) $$\sigma_{\text{movement}}^{LA_{1}}=\sigma_{\text{movement}}^{LA_{2}}={\left(\begin{array}{ccc} 0 & 0 & 0.1\text{rad} \end{array}\right)}^{T}.$$

Each limb has one degree of freedom and a length equal to 1. Results are averaged over 200 runs. In each run, the arm is initially set to a new random position, while the state estimates start with uniform distributions (i.e., no knowledge).

#### 3.1.1. Distribution of neurons

Both neurons and mappings are built once before starting all 200 runs. The angles **x**^*LA*_1_^ and **x**^*LA*_2_^ can take on values in the interval (−π, π) on the *z*-axis. The direction parts of the global (local) orientation **x**^*GD*_1_^ (**x**^*LD*_1_^) and **x**^*GD*_2_^ (**x**^*LD*_2_^), as well as the location of the elbow, are on the unit circle. Thus, the populations in the modules *LA*_1_, *LO*_1_, *GO*_1_, *GL*_1_, *LA*_2_, *LO*_2_, and *GO*_2_ all need to cover **lines** with the length 2π. Only the wrist location deviates from this: it must cover a whole **disk** with radius 2.

Two hundred Neurons are sampled in each of the former modules. Thus the average Euclidean distance between two neighboring neurons equals to

(27) $$d_{\text{avg}}=\frac{2\pi }{200}\approx 0.031$$

The minimum allowed distance between two neurons (cf. section 2.2.1) is set to *d*_min_ = 0.7 · *d*_avg_. In order to achieve the same average distance in *GL*_2_, the number *N*^*GL*_2_^ of neurons which need to be sampled is defined by

(28) $$\sqrt{\frac{\pi r^{2}}{N^{GL_{2}}}}=\frac{2\pi }{200}.$$

The *GL*_2_ neurons are distributed on a disc with radius *r* = 2 + 3σ^*GL*_2_^_Map_ = 2.09. The summand 2 accounts for the two limb lengths from shoulder to wrist, while 3σ^*GL*_2_^_Map_ (cf. section 3.1.2) guarantees that some neurons have receptive fields outside but close to the arm's reach. This slightly enlarged neural coverage avoids that boundary effects distort a probability distribution. The enforced equality (Equation 28) yields *N*^*GL*_2_^ = 14.0 · 10^3^ neurons.

#### 3.1.2. Mappings

We chose the standard deviation for the mapping's spreading (cf. Equation 16) so that it is equal to the average neuron distance, i.e., σ^*i*^_Map_ = *d*^*i*^_avg_ ≈ 0.031. The mappings spread radially, i.e., **R**^*i*^_Map_ = diag(σ_Map_), where diag refers to a diagonal matrix. We discarded any mappings that fall outside a 3σ_Map_-range.

#### 3.1.3. Tracking of information

In order to track the information influence stemming from one module (here *GL*_2_), we (1) introduced an offset to *GL*_2_ and (2) set its noise very low when compared to the other modules. The offset is introduced for two reasons: to distinguish the information that originates in *GL*_2_ from all other information, and to observe how nMMF reacts to the sudden failure of a sensor. The offset has a magnitude of 0.5 limb length. It is switched on at time *t* = 4 and switched off again at *t* = 7. The offset is in a counterclockwise direction (i.e., from the arm's perspective, the offset is to the left). *GL*_2_'s noise is low compared to other modules, in order to increase *GL*_2_'s impact. We chose radial Gaussians for the sensor noise:

(29) $$\sigma^{i}=\{\begin{array}{ll} 0.05\text{ limb length} & \text{if }i=GL_{2}\\ 0.5\;(\text{in rad},\text{limb length},\ldots ) & \text{otherwise} \end{array},$$

where σ is the standard deviation.

Evaluating nMMF when conflict resolution is applied allows us to determine whether the sensor failure can be detected and how well nMMF compensates for it. When conflict resolution is turned off, the setup shows how information starting in *GL*_2_ is generally propagated across modalities, frames of reference, and limbs.

### 3.2. Detection of sensor failure

**Figure 7:**
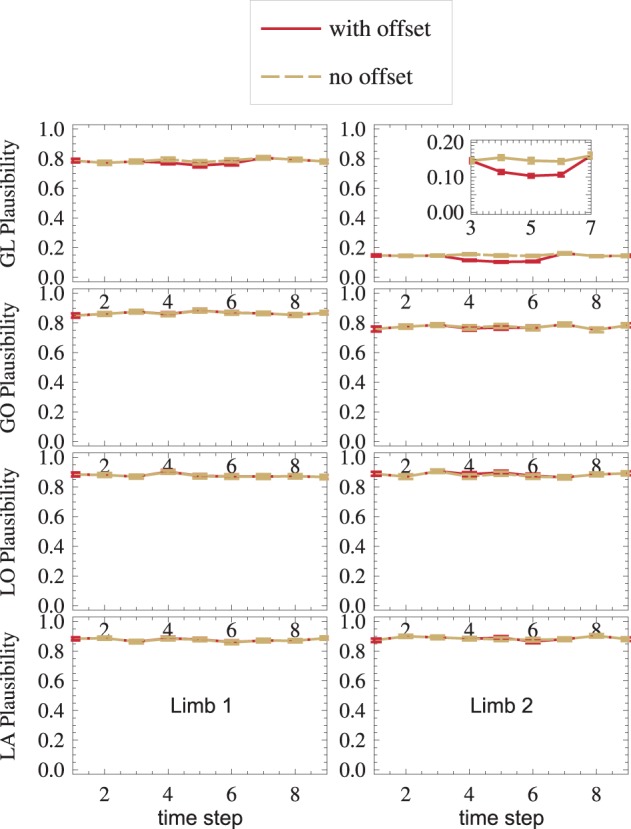
**Sensor failure is detected: in the *GL*_2_ module, where the sensor offset is introduced in time steps *t* ∈ [4,6], the plausibility drops**. Error bars are standard errors.

A sensor failure is modeled by the *GL*_2_-sensor offset during the interval *t* ∈ [4, 6]. By comparing all sensors, nMMF autonomously infers plausibility measures (Equation 22), which are displayed in Figure 7.

Even outside the offset-interval, *GL*_2_ (top right) shows a low plausibility *m* as compared to other modules. This is because, in general, three aspects characterize a distribution: its mean, its shape, and its dispersion. However, deciding which of these characteristics should be tested by a matching-measure *m* depends on the application. For instance, Equation (22) compares all three characteristics. As *Gl*_2_'s receptive field (Equation 29) is narrower than all other receptive fields, its dispersion is lower, and *m*^*GL*_2_^ mainly detects the different dispersions, while it might be more interesting to instead detect systematic errors of the mean. Thus, for this application, a dispersion-independent measure (Ehrenfeld and Butz, 2012, 2013) might be more appropriate. This would yield much higher measures *m*^*GL*_2_^ than shown in Figure 7, top-right.

Nevertheless, the measure is still able to detect sensor failure: while the offset is present (*t* ∈ [4,6]), the plausibility measure drops in the setup with offset (red), as compared to the setup without offset (yellow) (Figure 7, top-right).

### 3.3. Compensation of sensor failure

**Figure 8:**
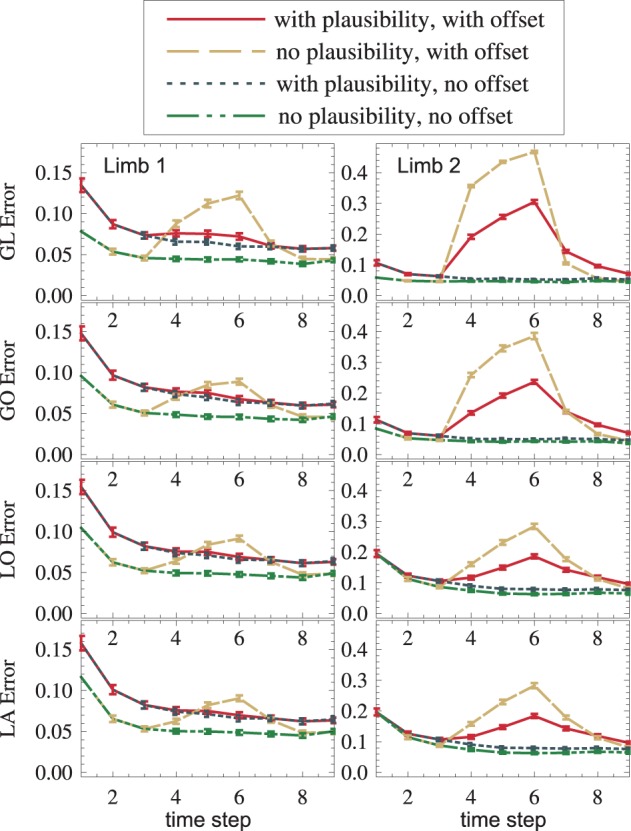
**An offset is propagated from *GL*_2_ to other modality frames and toward the upper arm (dashed yellow)**. The usage of plausibilities reduces the offset's influence (the solid red curve is lower than the dashed yellow curve).

Plausibilities were introduced as a measure of quality of an information source. If all sources provide correct data, plausibilities introduce a random change on otherwise Bayesian fusion. Such a change can only worsen the state estimate. The results confirm this: With plausibilities switched on, state estimates get worse (cf. red vs. yellow, blue vs. green in Figure 8). If, however, a sensory source is conflicting the others (red and yellow in the interval *t* ∈ [4,6]), plausibilities can suppress the influence of the false sensor information and improve the overall state estimate (red vs. yellow in Figure 8). This improvement is even visible under strong noise (red vs. yellow in Figure 8). Again, a dispersion-independent measure (Ehrenfeld and Butz, 2012, 2013) could improve the performance.

### 3.4. Propagation of information across modalities, frames of reference and limbs

The setup without conflict resolution (Figure 8, yellow and green) shows how information is propagated across modality frames and limbs in general. The yellow peak, which starts in *GL*_2_ (top right), is successfully propagated to all other modality frames (from top to bottom) and to the next proximal limb (from right to left). Shown is the estimation error (Euclidean distance between the real arm state and the estimated arm state).

### 3.5. Performance improvement due to distal-to-proximal mappings

**Figure 9:**
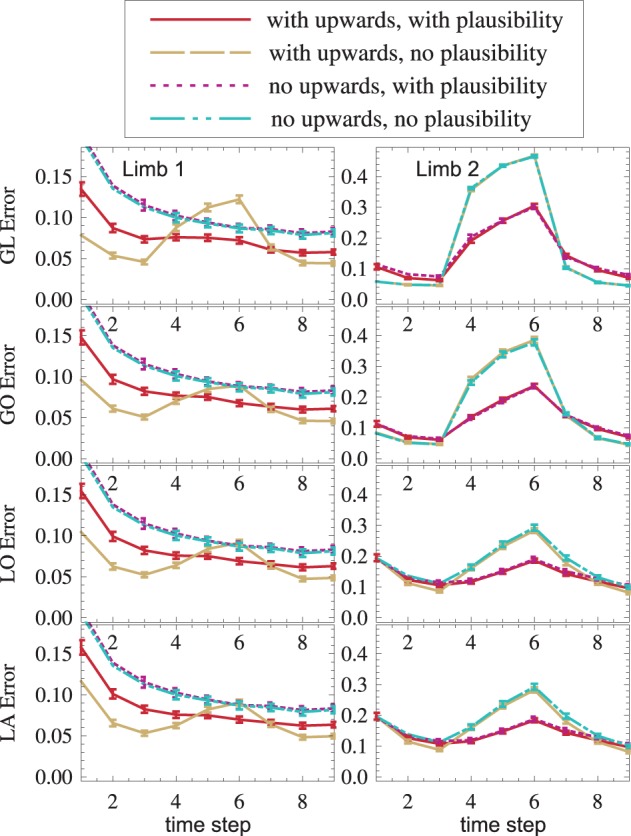
**(1) Distal-to-proximal mappings improve the state estimate (dashed yellow is lower than dash-dotted cyan, solid red is lower than dotted magenta). (2) Plausibilities worsen the state estimate if no failures exist and improve the state estimate if failures exist (red vs. yellow, magenta vs. cyan). Both effects (1) and (2) are found—though weaker—in other modules (not shown)**.

In order to see if distal-to-proximal mappings improve or worsen the state estimation, two setups, one with mappings and one without are compared. Figure 9 shows that the proximal limb's state estimate improves (yellow vs. blue, red vs. purple) because additional information flows to it from the distal limb. A slight improvement can even be seen in the distal limb. This is the case because the distal limb profits from more accurate forward and inverse kinematic estimates in the proximal limb.

## 4. Discussion

We introduced the neurally-encoded modular modality frame (nMMF) model, which maintains a consistent and robust but also highly distributed body state estimate over time. As in the previously published Gaussian MMF model (Ehrenfeld and Butz, 2011, 2012, 2013), nMMF represents the body (an arm in the current implementation) modularized into body parts and sensor-respective frames of reference. Local, body-state-dependent mappings allow for continuous interactions between modules, ensuring consistency. Bayesian information fusion principles are applied to fuse sensory information in the respective modules, to compare redundant information across modules, and to adjust the modular body state estimate for maintaining estimation consistency. Forward models are used to anticipate the sensory consequences of own movements and thus to fuse the consequent sensory information even more effectively.

In contrast to the MMF model, we showed that the same principles can be realized by means of a neural implementation, adding to the plausibility of the model. To succeed, population encodings principles of state estimates had to be employed. To establish a population code in one nMMF module, arm states were sampled randomly. To establish the neural mappings between the population codes, weight matrices were set based on the distances of the connected neurons, where the distances were currently determined by an informed kinematic model of the arm. To determine plausibility values, we used the scalar product to compare two neurally-encoded distributions. To avoid overconfidences in body states and to effectively realize information fusion, we normalized the resulting distributions maintaining respective Shannon entropies in the neural encodings.

In further contrast to the MMF model, nMMF also includes information exchanges from distal to proximal limbs and joints. This addition enables further-reaching information exchange. For example, information about the hand location can also influence estimates of the lower and upper arm, which was not the case in the MMF model (Ehrenfeld and Butz, 2013).

The evaluations confirmed that information from the wrist location influenced the whole arm estimate. First, we showed that due to the addition of the distal-to-proximal mappings, the location of the elbow or angles in the shoulder were adjusted by nMMF to generate an overall representation that is more consistent with the wrist estimate. We also showed that the additional mappings improve the state estimate due to the additional information exchange. Second, we showed that a systematic sensor error can be detected with the neural encoding. Third, although the inclusion of plausibilities slightly decreases the quality of the state estimate when all information sources are valid, if a sufficiently strong systematic error occurs in a sensor then the plausibility estimate can block this inconsistent information. Such sensor errors can be compared with situations in which visual information about the location of the hand is inaccurate, as is the case in the rubber hand illusion, thus leading to a misjudgment of the hand's location. The distal-to-proximal mappings in nMMF suggest, in addition to a misplacement of the hand, that the internal estimates of the elbow angles and lower arm orientations should be affected by the illusion.

### 4.1. Related models

The original motivation to develop the nMMF model came from SURE_REACH (Butz et al., 2007), a neural, sensorimotor redundancy resolving architecture, which models human arm reaching. SURE_REACH and the strongly related posture-based motion planning approaches (Rosenbaum et al., 2001; Vaughan et al., 2006) focused on flexible goal reaching capabilities and on anticipatory behavior capabilities, such as modeling the end state comfort effect (Rosenbaum et al., 1990). The current state of the body, although incorporated during action decision making, was not explicitly represented. In contrast, nMMF primarily focuses on the probabilistic, distributed representation of the body and effective information exchange. However, we believe that the nMMF model is ready to be combined with goal-oriented behavioral decision making, planning, and control routines. Moreover, while the SURE_REACH model was also implemented by neural grids, it represented the angular space of the arm in one module. Such a representation, however, is unfeasible for a seven degree of freedom, humanoid arm. nMMF's modularizations yield spatial encodings that are maximally three dimensional. Thus, nMMF is applicable to a seven degree of freedom arm. In particular, while SURE_REACH needs *O*(*x*^7^) neurons to cover the angular space of a humanoid arm with a density of 1/*x* neurons per dimension, nMMF only needs *O*(3*x*^3^) neurons to encode a comparable density.

The locality and modularity of nMMF relate the model to the mean of multiple computations (MMC) model (Cruse and Steinkühler, 1993; Schilling, 2011). However, nMMF additionally provides a probabilistic state representation, rigorous Bayesian-based information exchange, and plausibility-enhanced sensory information integration mechanisms. While the MMC model focuses on motor control, the nMMF model focuses on an effective, probabilistic body state representation. Nonetheless, the similarity to MMC suggests that similar motor control routines are implementable on a neural level in nMMF. Moreover, the fact that distributed, multisensory bodily representations serve well for goal-directed motor control (Andersen and Buneo, 2002) suggests that nMMF should be extended with adaptive motor control capabilities.

Various models use population codes for encoding probability distributions and exchange information in a comparable Bayesian fashion (Deneve and Pouget, 2004; Knill and Pouget, 2004; Doya et al., 2007). Information exchange across modalities and frames of reference take place in the brain. Gain fields are good candidates for realizing frame-of-reference conversions neurally (Andersen et al., 1985; Salinas and Abbott, 1995; Hwang et al., 2003; Deneve and Pouget, 2004). In the current nMMF implementation we used fully connected, direct transformations, which will need to be adjusted to gain-field transformations in order to map two three dimensional spaces into a third space. Nonetheless, in contrast to the related models, nMMF realizes a fully modularized, distributed probabilistic arm representation, which, to the best of our knowledge, has not been accomplished before. For example, Deneve and Pouget (2004) reviewed a multimodal gain field model that exchanged auditory, visual, and eye position information, enforcing consistency via population encodings. While nMMF has not considered auditory information so far, it goes beyond previous models in that it also incorporates a kinematic chain, relating body parts to each other along the chain. Thus, besides exchanging information across different frames of references, nMMF also exchanges information from distal-to-proximal body parts and vice versa.

In sum, nMMF focuses on estimating the own body state, incorporating multiple sources of information across sensory modalities and their respective frames of reference, as well as across neighboring body parts. While flexible goal-oriented behavior cannot be generated by nMMF at this point, the relations to the MMC model, the SURE_REACH model, and the posture-based motion planning theory suggest that behavioral decision making, planning, and control techniques can be incorporated.

### 4.2. Future work

Although the plausibility measure used in this work is generally well-suited, our previous work showed that a more rigorous normalization can yield very little information loss but the same gain in robustness when plausibilities are applied (Ehrenfeld and Butz, 2012, 2013). A similar normalization in the neural implementation seems to be possible only by means of heuristics, lacking the computational rigor. We are currently investigating alternatives.

In the current nMMF implementation several choices had to be made about which information should be exchanged, how plausibilities should be computed, and which reference frames should be represented. Additional frames-of-reference could be represented, such as a local location frame. Synergistic body spaces may also be represented, potentially accounting for the synergistic properties of the human body, the muscle arrangements, and the neural control networks involved (Latash, 2008). Also, plausibilities may be determined by considering the internal state estimations in addition to the redundant sensory information sources. Finally, the transformations between limbs and frames-of-reference may also be endowed with uncertainties. In this way, the body model itself would become adjustable, potentially accounting for illusions such as the Pinocchio illusion (Lackner, 1988), where a body part (e.g., the nose) elongates phenomenally.

Due to its modularity and focus on bodily representations, we believe that nMMF can be easily integrated into a layered control architecture. In such an architecture, other layers may encode extended bodily motion primitives, plan the desired kinematics of bodily motions, or control the dynamics of the body. In particular, extended motion primitives may be incorporated in order to execute a motion sequence, potentially selectively with any limb or joint currently available, similar to us being able to push down a door handle by means of our hands but also potentially with one of our elbows. Meanwhile, kinematic planning mechanisms may utilize the nMMF representation to generate motion plans online. Finally, lower-level dynamic control layers may be included.

## 5. Conclusion

In conclusion, this paper has shown that a distributed, probabilistic bodily representation can be encoded by modularized neural population codes based on Bayesian principles. The presented nMMF architecture is able to mimic the capability of humans to integrate different sources of information about the body on the fly, weighted by the respective information content. Bodily illusions can also be mimicked. Besides the more rigorous modeling of human data with nMMF beyond qualitative comparisons, we believe that nMMF should be embedded in a layered representation and adaptive control architecture in order to generate flexible and adaptive goal-oriented behavior.

### Conflict of interest statement

The authors declare that the research was conducted in the absence of any commercial or financial relationships that could be construed as a potential conflict of interest.

## Footnotes

^1^Note that Table 1 lists the terminology utilized in this paper.

^2^Without any additional specification, arm states are encoded in a “global”, i.e., shoulder-respective coordinate system. In the case of the local orientation (*LO*) modality frame, however, the coordinate system used to encode the state is relative to the next proximal arm limb. We use the pre-superscript to denote the encoding of a location in a limb-relative coordinate system. For example, ^*i*−1^**λ**_*i*_ denotes the location of limb *i* relative to the location **λ**_*i*−1_ and orientation of limb *i* − 1.

^3^In contrast, the fourth diagonal direction, distal-to-proximal-inverse, is not a single transformation step: the fingertip location and the hand orientation simply do not influence the proximal arm's orientation directly.

^4^Incomplete information: If e.g., a location input *GL* is transformed into the global orientation module *GO*, the result specifies only one subvector in the direction of the arm, while the other, perpendicular subvector remains unspecified. The second approach can then easily fuse a complete *GO* input onto this incomplete information.

^5^While *q* denotes the probability mass of a body state estimate, *s* denotes the probability mass of a neurons response to sensory input.

## Appendix

### A.1 Pseudoinverse and activation of a gaussian

A multivariate Gaussian with mean **μ** and Covariance-matrix **P** is given by:

(30) $$N\text{​}\left(\mu ,P\right)(x)\equiv \frac{1}{\sqrt{{\left(2\pi \right)}^{k}\left|P\right|}}{\text{e}}^{-\frac{1}{2}{\left(x-\mu \right)}^{T}P^{-1}\left(x-\mu \right)},$$

where *k* is the number of dimensions in the manifold.

In nMMF, three cases occur where activity is spread over neighboring neurons: when sensory inputs are encoded (Equation 6), when neural activity is propagated to other modules (Equation 16), and when the body estimate is updated with the movement (Equation 23). If the involved tuning functions are Gaussian, the activity mass spreads to all individual neurons *l* according to

(31) $$q_{l}\left(\mu ,P\right)\;=f\cdot \frac{V_{l}N\text{​}\left(\mu ,P\right)(x_{l})}{\sum_{l^{*}}V_{l^{*}}N\text{​}\left(\mu ,P\right)(x_{l^{*}})},$$

where **μ** is the new mean, **P** the tuning functions covariance, and *f* the activity mass, which is spread. For instance, if a sensory input is activated, **μ** is equal to the sensory reading and *f* is 1. If, on the other hand, the activity of a single neuron **x**_*n*_ is updated with a movement Δ**x**, **μ** is equal to **x**_*n*_ + Δ**x**, and *f* is equal to the neuron's probability mass *q*_*n*_.

If no inverse **P**^−1^ exists, it is approximated with the pseudoinverse **P**^+^. The pseudoinverse is computed via a singular value decomposition, which factorizes the (real) *m* × *m* covariance **P** into

(32) $$P=U\Sigma V^{T},$$

where **U** and **V** are unitary and Σ diagonal. **U** and **V** can be understood as rotation matrices while Σ is responsible for the scaling. Then the pseudoinverse **P**^+^ is

(33) $$P^{+}=V\Sigma^{+}U^{T},$$

where the pseudoinverse Σ^+^ of the diagonal matrix Σ is obtained by taking the reciprocal of every non-zero element. If a diagonal element of Σ is equal to zero, this has to be interpreted as the probability distribution not depending on that element. Consistent with that interpretation is Σ^+^: the corresponding diagonal element remains zero and deviations (**x**_*l*_ − **μ**) of the mean in the direction of that element are multiplied with zero, i.e., they do not lower the result of the Gaussian (Equation 31). Unfortunately, this is a singularity. For diagonal elements close but unequal to zero, Σ^+^ and consequently **P**^+^ explode. This occurs especially if a sub-manifold in a higher dimensional space needs to be activated (e.g., a sphere of elbow positions).

Thresholds are introduced to prevent discretization errors and small numerical errors from destabilizing the model. Matrix elements (*P*^−1^)_*ij*_ larger than the threshold 10^12^ are set to zero:

(34) $${\left(\Gamma \right)}_{ij}=\Theta \left(10^{12}-{\left(P^{-1}\right)}_{ij}\right){\left(P^{-1}\right)}_{ij},$$

where Θ is the heavyside function. Following, the distance vectors α_*l*_ and β_*l*_ are introduced as **x**_*l*_ − **μ** and Γ(**x**_*l*_ − **μ**) and bound by the threshold 10^−10^:

(35) $${\left(\alpha_{l}\right)}_{i}\;=\Theta \left({\left[x_{l}-\mu \right]}_{i}-10^{-10}\right)\cdot {\left[x_{l}-\mu \right]}_{i}$$

(36) $${\left(\beta_{l}\right)}_{i}\;=\Theta \left({\left[\Gamma \left(x_{l}-\mu \right)\right]}_{i}-10^{-10}\right)\cdot {\left[\Gamma \left(x_{l}-\mu \right)\right]}_{i}$$

Thus, Equation (31) becomes

(37) $$q_{l}\left(\mu ,P\right)\;=f\cdot \frac{V_{l}{\text{e}}^{-\frac{1}{2}\alpha_{l}^{T}\beta_{l}}}{\sum_{l^{*}}V_{l^{*}}{\text{e}}^{-\frac{1}{2}\alpha_{l^{*}}^{T}\beta_{l^{*}}}},$$

### A.2 Voronoi cell and voronoi volume

When *N* neurons are spread over a sample space Ω at positions **x**_*l*_, *l* ∈ (1..*N*), the Voronoi-cell *R*_*l*_ of a neuron *l* is defined as the set of all points *x* that are closer to the neuron position **x**_*l*_ than to any other neurons, i.e.,

(38) $$R_{l}\equiv \left\{x|\forall m:\left|\left|x-x_{l}\right|\right|\leq \left|\left|x-x_{m}\right|\right|\right\}$$

where ||·|| is the Euclidean norm. Intuitively, it is the subspace to which the neuron responds stronger than any other neurons. The Voronoi volume is defined as the volume of that cell. As only relative values are required, any normalization of the Volumes *V*_*l*_ is arbitrary.
